# Post-Transfusion Simultaneous Thrombotic Thrombocytopenic Purpura and Hemolytic Uremic Syndrome: A Rare Occurrence

**DOI:** 10.7759/cureus.30181

**Published:** 2022-10-11

**Authors:** Maharshi Patel, Twinkle Pawar, Sachin Agrawal, Gargi Mudey, Sunil Kumar, Sourya Acharya, Nishtha Manuja

**Affiliations:** 1 Department of Medicine, Jawaharlal Nehru Medical College, Wardha, IND; 2 Department of Medicine, Jawaharlal Nehru Medical College, Datta Meghe Institute of Medical Sciences, Wardha, IND; 3 Department of Microbiology, Jawaharlal Nehru Medical College, Wardha, IND

**Keywords:** plasmapheresis, acute kidney injury, thrombi, intravascular, endothelial

## Abstract

Endothelial cell injury, intravascular platelet-fibrin thrombi, and vascular damage are found in hemolytic uremic syndrome (HUS) and thrombotic thrombocytopenic purpura (TTP). The two disorders frequently manifest independently and are the important causes of acute renal damage. Acute kidney injury developed in our patient after blood transfusion and later on, the patient developed neurological complications. The patient was managed symptomatically and conservatively. Plasmapheresis and corticosteroid administration showed improved results.

## Introduction

Hemolytic uremic syndrome (HUS) and thrombotic thrombocytopenic purpura (TTP) are characterized by endothelial cell injury, intravascular platelet-fibrin thrombi, and vascular damage. The two illnesses commonly present separately and are the important causes of acute kidney injury. Blood transfusion can cause various adverse transfusion reactions like rash, itching, febrile non-hemolytic transfusion reaction, acute hemolytic transfusion reaction, septic transfusion reaction, transfusion-related acute lung injury, transfusion-associated circulatory overload, delayed hemolytic transfusion reaction, transfusion-associated graft versus host disease, and post-transfusion purpura [1]. One recently reported TTP/HUS case involved a patient who underwent simultaneous pancreas/kidney (SPK) transplant followed by pancreas rejection and clopidogrel treatment. One combined TTP/HUS case had been reported in neuromyelitis optica [2].

In this case report, we highlight simultaneous TTP/HUS in a 20-year-old female who presented with acute kidney injury after a blood transfusion. After a literature search, this case report is probably the first in the world.

## Case presentation

A 20-year-old female patient presented to the hospital with the chief complaint of vomiting (five episodes) and blood in her urine for seven days. She also complained of fever, burning micturition, and heavy menorrhagia. There was no history of similar complaints in the past. She denied any history of diabetes mellitus, systemic hypertension, tuberculosis, bronchial asthma, or thyroid disorders.

On examination, the patient was afebrile, pulse was 76/min and regular, and blood pressure was 100/70 mm Hg. On general examination, pallor was present. On systemic examination, the cardiovascular system and respiratory system were normal; the examination revealed normal s1 s2, and no murmur was heard. Respiratory system examination revealed bilateral breath sounds with no adventitious breath sounds. On central nervous system examination, the patient was conscious, and oriented to time, place, and person; the abdomen was soft and non-tender.

The patient was taken to a private hospital where she was transfused with three units of whole blood in view of severe anemia. Post transfusion, the patient developed decreased urine output and altered kidney function test (KFT).

**Figure 1:**
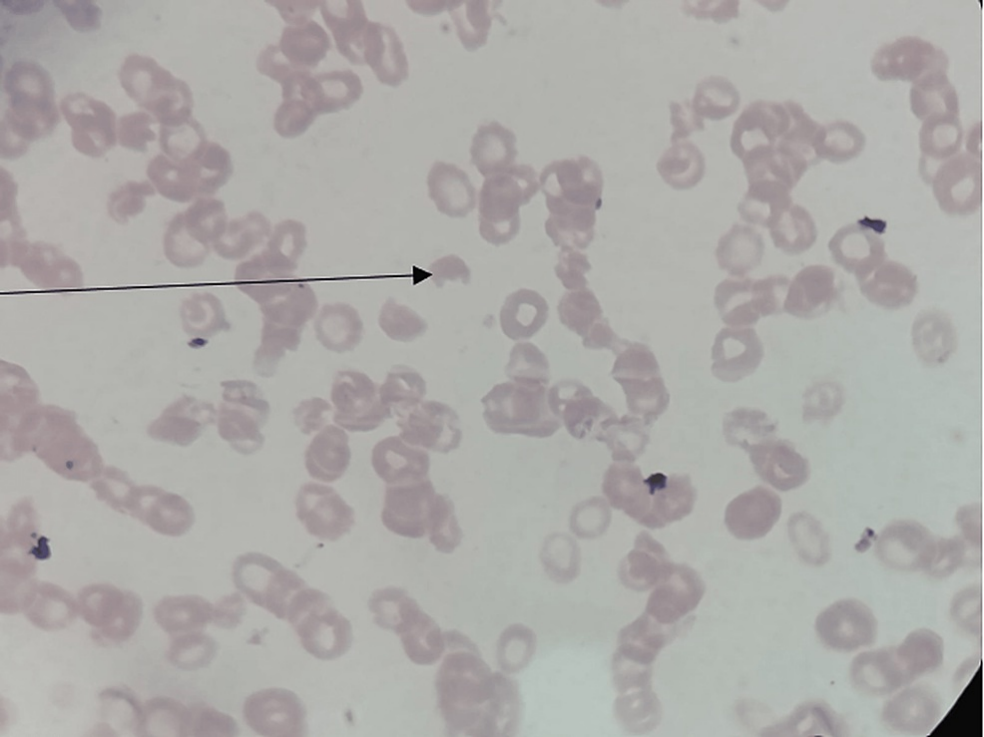
Schistocytes in peripheral smear (black arrow)

**Figure 2:**
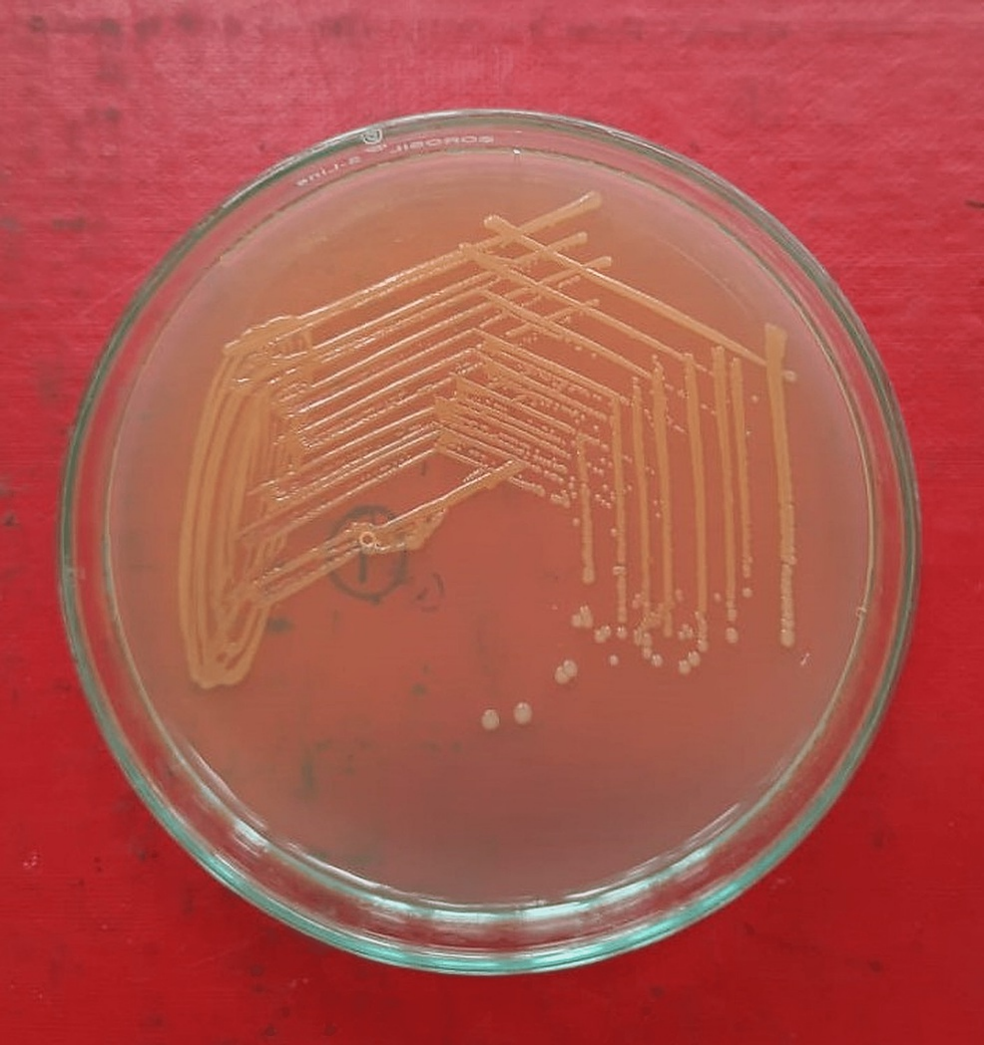
Growth of Escherichia coli bacteria in culture medium

The patient's laboratory reports are shown in Table 1. A complete blood count (CBC) was suggestive of severe anemia and thrombocytopenia. Peripheral smear was suggestive of the presence of schistocytes (Figure 1). Urine routine microscopy was suggestive of dark reddish brown color urine containing 4-5 red blood cells per high power field (RBC/HPF). Liver function test was suggestive of unconjugated hyperbilirubinemia. KFT was suggestive of increased urea and creatinine. Sickling and Coomb’s test was negative. Urine culture was suggestive of the growth of Escherichia coli (E. coli) (Figure 2).

**Table 1:** Lab values of the patient

Laboratory Test	Value
Hemoglobin	4.5 g/dl (normal range: 12-15 g/dl)
Mean Corpuscular Volume	80 fl (80-100 fl)
Platelets	67000/cumm (1.5 lacs - 4 lacs/cumm)
White blood cells	5400/cumm (4000-11000/cumm)
Prothrombin Time	12.0 seconds
International Normalized Ratio	1.00
Activated Partial Thromboplastin Time	29.9 seconds
D-dimer	584 ng/ml (0-255 ng/ml)
Erythrocyte Sedimentation Rate	54 mm (3-15)
Urea	163 mg/dl (7-17 mg/dl)
Creatinine	10.6 mg/dl (0.52-1.04 mg/dl)
Sodium	143 mmol/L (135-145 mmol/L)
Potassium	4.0 mmol/L (3.5-5.5 mmol/L)
Lactate Dehydrogenase	2655 U/L (120-246 U/L)
Alkaline Phosphatase	48 U/L (40-126 U/L)
Alanine Transaminase	26 U/L (<35 U/L)
Aspartate Transaminase	101 U/L (14-36 U/L)
Albumin	3.6 g/dl (3.5-5 g/dl)
Total Bilirubin	2.9 mg/dl (0.2-1.3 mg/dl)
Unconjugated	1.5 mg/dl (0-1.1 mg/dl)
Conjugated	0.7 mg/dl (0-0.3 mg/dl)

The patient had one episode of seizure during the hospital stay. Random blood sugar was normal and there was no fever spike. MRI brain was suggestive of a normal study.

The patient was managed conservatively and was started on hemodialysis, plasmapheresis, antibiotics, blood transfusion, and corticosteroid. After initiating the treatment, the patient improved; urine output increased, urea and creatinine levels decreased, and urine color improved.

## Discussion

TTP is characterized by a pentad of microangiopathic hemolytic anemia, thrombocytopenia, renal failure, neurologic deficits, and fever. The full-blown condition is becoming less prevalent, most likely due to early detection. Primary or acquired TTP is usually differentiated based on ADAMTS13 which is deficient due to mutation and due to autoantibodies, respectively. Its deficiency is caused by an IgG autoantibody and should be considered an autoimmune disorder [3]. Unfortunately, ADAMTS13 was not done in our patient due to financial constraints, so we were unable to determine if the TTP was primary or acquired.

In addition, we could not differentiate TTP from HUS in this case. The triad of HUS consists of acute renal failure, microangiopathic hemolytic anemia, and thrombocytopenia. Although renal damage is more frequent and prominent in HUS, central nervous system damage has also been reported in HUS. Hence, the differentiation between HUS and TTP can be challenging and difficult [3]. In our patient, neuronal involvement was in the form of one episode of generalized seizure, a reason we could not explain.

Our patient had acute kidney injury which occurred post blood transfusion, but there were no laboratory reports from outside the hospital suggestive of HUS/TTP. The exact pathophysiological mechanism of transfusions-induced acute kidney damage is unknown; it is known that erythrocytes undergo irreversible morphological and metabolic changes during storage. As a result, they can create a pro-inflammatory state after transfusion, decrease tissue oxygen transport, and worsen tissue oxidative stress. This in turn can cause AKI in susceptible patients undergoing cardiac surgery with cardiopulmonary bypass, such as those with pre-existing kidney dysfunction or anemia [4-6].

Most commonly, HUS developed after a gastrointestinal illness called diarrhea-associated HUS due to the close linkage between infections with Shiga toxin-producing strains of E.coli. But in this case, HUS/TTP was due to E. coli infection in urine confirmed by the culture report. There is a significant difference in the pathophysiology of the Shiga toxin E. Coli, HUS, atypical HUS, and TTP, but the clinical manifestation is more or less similar [7]. In our case, the patient developed simultaneous HUS/TTP post transfusion; the patient was managed by detecting the disease early and was given early dialysis which eventually improved the prognosis.

TTP, immune thrombocytopenic purpura (ITP), and disseminated intravascular coagulation (DIC) share some similarities but their pathogenesis is different. ITP is caused by antiplatelet antibodies, TTP is caused by endothelial defects and DIC is caused by thrombin excess. Schistocytes can be found in both DIC and TTP but they are absent in ITP. The coagulation profile is deranged mainly in DIC. In DIC, there is decreased fibrinogen and raised fibrin monomer, fibrin degradation, and D-dimer. Steroids, IVIg, and splenectomy is the mainstay treatment for ITP. Whereas, treatment of TTP consists of plasma exchange and vincristine. In DIC, plasmapheresis and antithrombin III (ATIII) can be useful [8].

Evens et al. reported the case of SPK transplant recipients developing TTP/HUS after receiving clopidogrel and sirolimus [9]. Gupta et al. published a study in which they found out that pregnancy-associated atypical HUS typically manifests in the postpartum period, frequently following a pregnancy complication, and eculizumab is successful in attaining disease remission [10]. Godhiwala et al. described HUS in a snake bite patient who had acute kidney injury, thrombocytopenia, and microangiopathic hemolytic anemia with a normal coagulation profile which was improved after plasmapheresis and dialysis [11].

## Conclusions

The combined atypical HUS and TTP occurrence after blood transfusion in a young patient is infrequent. In most cases, the therapy consists of resolving the anomaly that caused the disease, along with intensive care. Hence, the early diagnosis of atypical HUS and TTP in patients after blood transfusion can result in the early initiation of the treatment, which leads to a better prognosis for the patient and can reduce the morbidity and mortality of the patient. The research on this disease will provide us with a better diagnostic approach and treatment modalities.
